# *Periplaneta americana* extract promotes intestinal mucosa repair of ulcerative colitis in rat^1^

**DOI:** 10.1590/s0102-865020200100000002

**Published:** 2020-11-23

**Authors:** Nan-nan Xue, Miao He, Yue Li, Jun-zhu Wu, Wen-wen Du, Xiu-mei Wu, Zi-zhong Yang, Cheng-gui Zhang, Qi-yan Li, Huai Xiao

**Affiliations:** I Master, Yunnan Provincial, Key Laboratory of Entomological Biopharmaceutical R&D, Dali University, Yunnan, China. Acquisition of data, manuscript preparation.; II PhD, National-Local Joint Engineering Research Center of Entomoceutics, Dali University, Yunnan, China. Critical revision, final approval.; III Master, The People's Hospital of Linqing, Shandong Province, China. Acquisition, analysis and interpretation of data.; IV PhD, Yunnan Provincial, Key Laboratory of Entomological Biopharmaceutical R&D, Dali University, Yunnan, China. Conception and design of the study, manuscript writing, final approval.; V PhD, The First People's Hospital of Yunnan Province, Kunming, Yunnan Province, China. Histopathological examinations, critical revision, final approval.

**Keywords:** Periplaneta, Oxazolone, Colitis, Ulcerative, Interleukin-13, Epidermal Growth Factor, Rats

## Abstract

**Purpose:**

To investigate the mechanism of Periplaneta americana extract promoting intestinal mucosal repair of OXZ-induced colitis in rat.

**Methods:**

All experiments used an equal number of male and female SD rats (n=48). We injected OXZ into the colon to induce UC rat model. To determine the optimal concentration of P. Americana's extract (PA-40), it was classified into low (L), medium (M), and high (H) doses. After OXZ treatment, each drug was administered by enema for 7 consecutive days. Rats were divided into the following 6 groups: (1) Saline treatment group (NC), (2) OXZ treatment UC model group (MC), (3) OXZ + budesonide group (BUN), (4) OXZ + PA-40 L group, (5) OXZ + PA-40 M group, (6) OXZ + PA-40 H group. Disease activity index (DAI) scores, colon length, histopathological score, serum cytokine level (IL-4, IL-10, iNOS, tNOS), and amount of MPO, EGF, IL-13 in colonic mucosa were measured.

**Results:**

PA treatment had a significant healing effect on the OXZ-colitis model and significantly reduced the lesioned area, especially in the PA-40H groups. PA treatment did not alter the expression of IL-10 and MPO level, but increased EGF (epidermal growth factor) and decrease IL-13 in the colonic tissue. PA inhibited the rise of NOSs (nitric oxide synthase) and decreased the serum IL-4 level.

**Conclusions:**

The data suggest that Periplaneta americana extract may be a potential compound for the treatment of colonic lesions. The mechanism may be related to inhibiting the secretion of IL-13 and promoting the formation of EGF.

## Introduction

Inflammatory bowel disease (IBD) is a group of inflammatory diseases that affect the gastrointestinal tract, including Crohn's disease (CD) and ulcerative colitis (UC)^1^. UC is characterized by continuous inflammation of the lamina propria of the colon, accompanied by mucosal barrier damage and infiltration of inflammatory factors^2^. Clinical symptoms are abdominal pain, diarrhea, weight loss, mucopurulent blood stools and some common complications such as colon cancer^3^. It is the most common form of inflammatory bowel disease worldwide^4^. Although there has been significant progress in understanding the pathogenesis of IBD in the past few years, the main cause of IBD remains unclear. Environmental factors, immune factors and genetic susceptibility seem to promote the development of intestinal inflammation to a certain extent^5^ ^-^ ^9^. There are no treatments for reducing inflammation and restoring intestinal barrier function^10^. Commonly used clinical drugs for the treatment of UC include 5-aminosalicylic acid, corticosteroids, thiopurines, calcineurin inhibitors, anti–tumor necrosis factor (TNF) agents, antiadhesion molecules, JAK inhibitor^11^ ^-^ ^13^ and other drugs^14^. However, most drugs have some side effects or limited therapeutic effects. Oxazolone (OXZ), as a hapten, has shown to induce contact allergic reactions in all parts of the animal body. Studies at home and abroad have shown that its induced colitis is similar to human UC in histological features and inflammatory distribution^15^ ^,^ ^16^.

Insects are the world's largest animal population with over 700.000 species^17^. More than 1,900 edible insects have been identified worldwide, including crickets, locusts, mealy larvae, ants, grasshoppers and others, which contain high-quality proteins, minerals, fatty acids, vitamins and amino acids for human consumption^18^ ^,^ ^19^. The consumption of insects may have positive effects on human health. For example, cricket powder may stimulate the growth of intestinal microbiota, reduce the level of TNF-α in plasma, and it is rich in chitin and chitosan, which can inhibit pathogenic microorganisms in the intestines^20^ ^,^ ^21^. Locust powder can inhibit intestinal lipid absorption by changing the content of short-chain fatty acids in rat cecum^17^. As a kind of medicinal and edible insect, *Periplaneta americana* has been recorded in many important classical documents of traditional Chinese medicine (TCM) such as “shen nong Ben cao jing”^22^. Modern medical research has found that *Periplaneta americana* has anti-tumor^23^, enhanced immunity, antibacterial, anti-inflammatory and analgesic effects, tissue repair and so on^24^. Our previous study found that it has a good therapeutic effect on ulcerative colitis, but its mechanism of action is still unclear. As an extract of *Periplaneta americana*, PA-40 has been widely used in the treatment of ulcerative colitis in China, and has achieved good results. In this study, the therapeutic effect of PA-40 on OXZ-induced ulcerative colitis in rats was investigated, and the mechanism of action was preliminarily explored.

## Methods

### Reagents

Oxazolone (4-ethoxymethyl-2-phenyl-2-oxazolinone-5-1) was purchased from China shenzhen regent biochemical technology CO. LTD (Shenzhen, China). The occult blood kit, MPO (myeloperoxidase), NOS test kits and rat interleukin-13 ELISA kit were purchased from jiangsu nanjing jiancheng biotechnology CO. LTD (Nanjing, China). Rat interleukin-4 (IL-4), interleukin-10 (IL-10) and epidermal growth factor (EGF) ELISA kits were purchased from xinbosheng biotechnology CO. LTD (Shenzhen, China).

### Preparation of PA-40 in Periplaneta americana extract

*Periplaneta americana* was dried, crushed, sifted with 20 mesh, soaked in 80% ethanol solution of 10 times the amount for 2 h, then heated at 70°C for 4 h, extracted twice, combined with filtrate, and then evaporated for concentration. After D101 macroporous resin was applied, the column volume was eluted with pure water and then eluted with 40% ethanol. The 40% ethanol elution part was collected and concentrated into a viscous extract to obtain *Periplaneta americana* extract PA-40.

### Experimental animals

Sprague-Dawley rats (180-220g, equal ration of male and female) were obtained from Hunan SJA Laboratory Animal CO. LTD. The experiments follow the rules of the school's animal ethics committee, which approves all experiments for the purpose of controlling and supervising animal experiments (protocol number 2015-0820).

### Establishment of UC model

OXZ induced colitis method has been described by Heller etc^25^; the back skin of rats is shaved (2 cm × 2 cm), and shaving drops of 0.2 mL 3.0% OXZ solution (dissolved in anhydrous ethanol) are used for seven days; after 7 days, a suitable amount of isoflurane anesthesia is used with each rat, and then a 2 mm diameter polyethylene tube is inserted into the large intestine about 8 cm, with 1.0% of OXZ solution (dissolved in 50% ethanol) 1.0 mL·kg^−1^. The inversion of rats is performed 1 min after injection to prevent backflow.

### Experimental grouping

Normal control group (NC) was given normal saline (Enema, 2.5 ml·kg^−1^), and the model rats were stratified with DAI score according to Hamamoto etc^26^. After removing the rats with very mild inflammatory model, they were randomly divided into model group (MC), BUN group, and three groups with low, medium and high doses of PA-40, with 8 rats in each group. Rats in the MC group were given normal saline (Enema, 2.5 mL·kg^−1^) and rats in the budesonide group (BUN) (Enema, 0.2 mg·kg^−1^) were given three different doses of PA-40 (Enema, 50, 100 and 200 mg·kg^−1^, labeled as PA-40 L, PA-40 M, PA-40 H). Daily weight, fecal viscosity, and blood stools of all rats were recorded for 7 days after administration, and OXZ-induced colitis was assessed using the disease activity index (DAI) scoring system.

### The disease activity index

The daily clinical assessment of the rat after the challenge included measurement of body weight and evaluation of stool consistency and the presence of blood in the stools by occult blood kit, which was graded according to Table 1.

**Table 1:** DAI scoring system.

Score	Weight loss(%)	Stool consistency	Hematochezia
0	0	Normal	Normal
1	≥1 - <5	Semi loose (+)	Feces with Occult blood (+)
2	≥5 - <10	Semi loose (++)	Feces with Occult blood (++)
3	≥10 - <15	Loose (+)	Bloody feces (+)
4	≥15	Loose (++)	Bloody feces (++)

### Macroscopical evaluation of colitis colon

All rat colon tissues were rapidly stripped and their natural extension length was measured. After the colon was removed, the intestinal contents were flushed with sterile saline. The water was dried with a sterile blotting paper and the wet weight of the colon was measured. The colon mucosal damage index (CMDI) was graded according to Table 2.

**Table 2:** CMDI rating criteria.

Score	CMDI scoring criteria
0	no damage
1	mild hyperemia edema, smooth surface, no erosion or ulcer
2	Hyperemia and edema, rough mucosa, granular, erosion or intestinal adhesion
3	High hyperemia and edema, necrosis and ulceration on the surface, area < 1 cm^2^, intestinal wall thickening or necrosis and inflammatory polyps on the surface
4	Severe congestive edema, mucosal necrosis and ulceration, ≥ 1 cm^2^ or necrosis of the entire intestinal wall, toxic megacolon leading to death

### Histopathological evaluation

After the colon was macroscopical evaluated, it was fixed with 10% buffered formaldehyde, embedded in paraffin, sectionalized, and stained with hematoxylin and eosin (HE). Sections of colon tissue were analyzed under a microscope and photographed for preservation. The histopathological score was graded according to Table 3.

**Table 3:** Histopathological score.

Score	Epithelial cell	Inflammatory cell infiltration
0	Normal	no infiltration
1	Cup cell loss	Infiltrate into the basal layer of the crypt
2	Large areas of goblet cell loss	Infiltrates into the muscularis mucosa
3	Loss of crypt cells	Infiltration extends deep into the muscularis mucosa, accompanied by thickening of the mucosa and marked edema
4	Extensive loss of crypt cells	Infiltration reaches the submucosa

### Measurement of IL-13, MPO and EGF levels in colonic tissue

Colonic tissue was suspended in ice-cold PBS and then simply homogenized. The homogenate was centrifuged at 12000 rpm, 4°C for 10 min, and the supernatant was collected. The contents of IL-13 and epidermal growth factor (EGF) were determined by enzyme-linked immunosorbent assay. The content of MPO in colon tissues was determined as per kit requirements.

### Determination of IL-4, IL-10, iNOS and tNOS in blood serums

The whole blood samples of all rats were collected. They were centrifuged immediately at 12000 g, 4 °C for 10 min; then, the supernatant was collected and measured at −20 °C. The contents of IL-4 and IL-10 were determined by elisa kit, and the operation was carried out according to the kit instruction's requirements. Nitric oxide synthase (NOS) in the serum was measured according to the kit instructions.

### Statistical analysis

All experiment results were expressed as mean ± standard deviation (SD). One-way analysis of variance (ANOVA) and post-hoc analyses were used for the analysis of differences between groups. The SPSS 20.0 and GraphPad Prism software were used to generate a P-value and statistic for each analysis; P-value less than 0.05 was considered statistically significant.

## Results

### Ingredients of PA-40

**Figure 1:**
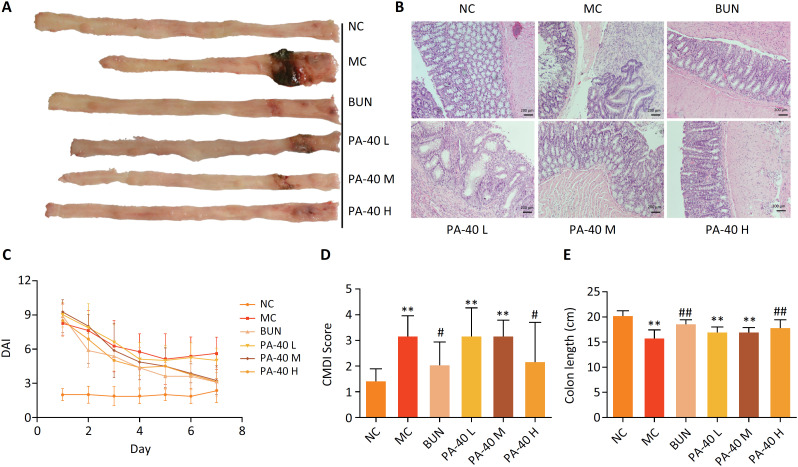
Morphological analysis of OXZ-induced colitis rat treated with PA-40. **(A)** Samples of macroscopic findings of colonic mucosa. OXZ treatment group (MC) showed obvious bleeding, hyperemia and edema. **(B)** Representative histopathological images of rat colorectum (scale bar: 200 *μ*m). MC group showed the loss of goblet cells, crypts and severe infiltration of inflammatory cells. **(C)** Time-dependent changes of DAI scores for 7 days by PA40 and BUN treatment. **(D)** The colon mucosal damage index (CDMI) of colon tissue samples. PA-40 H group significantly reduced the score compared with MC group. **(E)** The comparison of colon length between 6 groups. PA-40 treatment significantly ameliorated the colon length compared with MC group. Data are presented as mean ± SD of 8 rats in each group (**, P < 0.01 *vs*. NC group;^#^, P < 0.05, ^##^, P < 0.01 *vs*. MC group).

The polypeptide content was carried out by Lowry method using bovine serum albumin as a standard. The PA-40 polypeptide content was 51.54% (g/g) by the Lowry method. After the HPLC analysis of the chemical composition of PA-40, 5 compounds were identified in PA-40. They were uracil, hypoxanthine, uridine, adenosine and inosine (Fig. 1).

### Disease activity index (DAI)

A combinatorial DAI, considering percentage body weight loss, stool consistency, and bloody stool was used to evaluate the activity of the disease and the response to treatment. After 7 days of continuous enema, different groups of rats showed different degrees of enteritis symptoms. Compared with the NC group, the DAI score of rats in the MC group increased significantly. PA-40 L, PA-40 M and PA-40 H groups reduced DAI scores. But the treatment effect of the PA-40 H group (200 mg.kg^−1^) was better than that of the two other dosages (Fig. 1C).

### Macroscopical evaluation of colitis colon

As shown in Figure 1A, OXZ enema induced gross colonic mucosal injury, such as hyperaemia, oedema, wall thickness, necrosis, and ulceration compared to the control group that showed no mucosal damage (MC). These observations were similar to pathological changes in human IBD. Enema with BUN and PA-40 can significantly improve the symptoms of colonic mucosal injury (Fig. 1D). The length of the colon was significantly shortened by OXZ, and the length of the colon in the model group (15.63 ± 1.77 cm) was significantly shorter than that in the NC group (20.04 ± 1.22 cm) (Fig. 1A, E). Compared with the model group, the colon length of rats in the PA-40 treatment group was increased. But only the PA-40 H group (17.63 ± 1.74 cm) showed significant differences from the model.

### Histopathological evaluation

The NC group had complete crypt structure and epithelial cell layer, and the goblet cell morphology was normal. In the MC group, the lamina propria was damaged, epithelial cells were lost, inflammatory cells were strongly infiltrated, large areas of intestinal crypts were necrotic, bleeding and edema were observed, and neutrophils were severely infiltrated and penetrated to the muscle layer. The mucosa and crypt regenerated in BUN group, and the inflammatory response was decreased. In the PA-40 L group, the lamina propria recess disappeared, with superficial mucosal ulcer and inflammatory cell infiltration. PA-40 M group showed crypt dilatation, deformation and lamina propria fibrosis. PA-40 H group had normal recess structure and no inflammatory characteristics (Fig. 1B).

### Analysis of cytokine levels in colonic tissue

**Figure 2:**
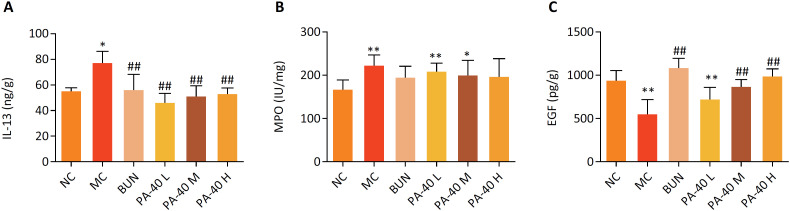
Anti-inflammatory and cell proliferation effects of PA-40 in OXZ-induced colitis rat. The expression levels of IL-13 **(A)**, EGF **(C)** were determined by ELISA kit. MPO **(B)** levels in colonic tissues were determined by MPO kit. Data are presented as mean ± SD of 8 rats in each group (*, P < 0.05, **, P < 0.01 *vs*. NC group; ^#^, P < 0.05, ^##^, P < 0.01 *vs*. MC group).

The expression levels of IL-13 and MPO in the colonic tissues of MC rats were significantly up-regulated, compared with the NC group. After PA-40 treatment, the expression levels of IL-13 and MPO in colon tissues were significantly reduced (*P < 0.05, **P < 0.01; ^##^ *P* < 0.01, Fig. 2A, B). It is suggested that PA-40 has certain anti-inflammatory effect on oxazolone-induced colitis. The colonic tissue EGF activity of rats in the MC group was significantly reduced, compared with the NC group. The activity of EGF in colon tissues of PA-40 M and PA-40 H group was significantly increased compared with that of MC group (**P < 0.01; ^##^ *P* < 0.01, Fig. 2C).

### Analysis of cytokine levels in blood serums

**Figure 3:**
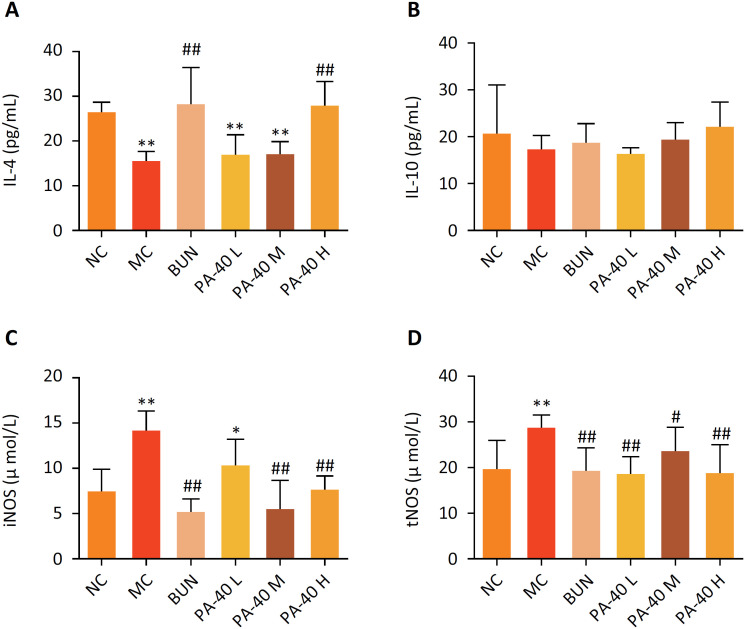
Anti-inflammatory effects of PA-40 in OXZ-induced colitis rat. The expression levels of IL-4 **(A)**, IL-10 **(B)**, were determined by ELISA kit. iNOS **(C)**, tNOS **(D)** levels in blood serums were determined by NOS kit. Data are presented as mean ± SD of 8 rats in each group (*, P < 0.05, **, P < 0.01 *vs*. NC group; ^#^, P < 0.05, ^##^, P < 0.01 *vs*. MC group).

The expression level of IL-4 in the MC group significantly decreased, compared with the NC group, (**P < 0.01, Fig. 3A), and IL-10 decreased slightly (**P > 0.05, Fig. 3B), but there was no statistical difference. The expression level of IL-4 in the PA-40 H group was significantly increased, compared with the MC group (^##^ *P* < 0.01, Fig. 3A). The expression level of IL-10 was slightly increased (Fig. 3B). The expression levels of iNOS and tNOS in the MC group significantly increased, compared with the NC group (**P < 0.01, Fig. 3C, D), and the expression levels of iNOS in the PA-40 M and PA-40 H groups significantly decreased (^##^ *P* < 0.01, Fig. 3C).

## Discussion

At present, the methods for establishing animal models of IBD mainly include spontaneous animal model, genotype animal model, cell transplantation animal model and chemical drug induction animal model, among which chemical drug induction is the most extensive. In our previous experiment, Dinitrochlorobenzene (DNCB) combined with acetic acid was used to induce the rat colitis model. DNCB is a hapten chemical, which induces delayed intestinal mucosal response and imbalance of Th1/Th2 immune cells in rats after repeated sensitization^27^. Acetic acid can increase intestinal mucosal vascular permeability, activate kinin, interfere with blood coagulation, and initiate inflammation, thus forming local inflammatory lesions^28^. OXZ-induced colitis is a typical model of Th2 colitis associated with immunity^29^. Due to its simple and repeatable properties, the induced colonic intervention model is widely used to screen potential therapeutic drugs and the model is similar to human UC, which can be used for further study of the disease.

In OXZ colitis, IL-4 is the initial cytokine produced by oxazolone colitis, but IL-4 is soon replaced by the production of IL-13, which can activate epithelial cells to secrete mucus and fluid^25^. This cytokine and IL-4 can lead to the destruction of tight connections between epithelial cells, which in turn opens the door to bacterial invasion. When IL-13 was neutralized, oxazolone administration did not cause any histological changes or other inflammatory symptoms^30^. This strongly suggests that IL-13 mediates mucosal tissue damage as an effector cytokine^31^. The expression level of IL-4 in serum of BUN and PA-40 group was significantly increased, and the level of IL-13 was significantly decreased. It is suggested that *Periplaneta americana* extract may promote the repair of intestinal mucosa by inhibiting the expression of IL-13 in UC rats^32^. IL-10, mainly secreted by Th2 cells, is an anti-inflammatory immune factor with immunomodulatory and anti-inflammatory effects, and plays an important role in regulating intestinal immune balance^33^. IL-10 showed no significant changes after treatment of BUN and PA-40. This may be because of the different sampling sites, or BUN and PA-40 inhibited Th2 responses.

Intestinal mucosal barriers include mechanical barriers, chemical barriers, immune barriers and biological barriers^34^. Epidermal growth factor (EGF) is an important cell growth factor that plays an important role in maintaining the intestinal mucosal barrier^35^ ^,^ ^36^. Supported by EGF, gastrointestinal mucosa has a significant ability to repair damage, and EGF stimulates cell migration and extracellular matrix formation^37^. EGF promotes wound healing by improving the integrity of intestinal epithelial and mucosal barriers in rats. The results of this experiment showed that BUN, PA-40 M and H dose groups could significantly improve the expression level of EGF.

Myeloperoxidase is a 140-kDa heme protein secreted primarily by neutrophils and stored in eosinophils^38^ ^,^ ^39^. Colonic injury leads to the activation of neutrophils and macrophages, and the release of MPO from activated neutrophils causes hydrogen peroxide (H_2_O_2_) to produce hypochlorous acid (HOCl) and chlorine anions (Cl^−^) to further damage the colon^40^. The activity of MPO is often used as an indirect indicator of tissue severity. In this study, BUN and PA-40H group could reduce the activity of intestinal mucosal MPO and reduce inflammatory response. But there was no significant difference. Physiological dose of NO can protect the digestive system, but excessive NO can stimulate the digestive system and lead to UC^41^. NOS is a rate-limiting enzyme for the synthesis of NO, which can indirectly reflect the level of NO. iNOS continuously release a large amount of NO after a certain stimulation can aggravate the degree of inflammation of the colon, and the higher the content of iNOS, the more serious the inflammation of UC. The results of this study suggested that in the OXZ-colitis model group, iNOS expression and NO release were increased, and NO and iNOS were positively correlated with MPO activity. After drug treatment, BUN, PA-40 treatment group of NO, iNOS expression significantly decreased. These results further confirmed that the extract of *Periplaneta americana* PA-40 has a certain therapeutic effect on OXZ-induced colitis.

## Conclusion

This study suggested that the therapeutic effect of PA-40 on UC may be realized by regulating the expression of related immune regulatory factors, inhibiting the expression of pro-inflammatory cytokines, and participating in promoting the repair of intestinal mucosa.
